# Text-Book of Hygiene

**Published:** 1891-08

**Authors:** 


Text-Book of Hygiene. A Comprehensive Treatise on the Principles and Practice of Preventive Medicine from an American Standpoint. By George H. Rohé, M. D., Professor of Obstetrics in the College of Physicians and Surgeons, Baltimore; Member of the American Public Health Association; Foreign Associate of the Société Francaise d' Hygiene, etc. Second edition, thoroughly revised and largely re-written, with many illustrations and valuable tables. 8vo, pp. x.—421. Philadelphia and London: F. A. Davis. 1890. Price, $2.50 net.

The author of this book attempts the impossible, namely, to make of one work a text-book and a comprehensive treatise. It is a very good text-book, but it is not, by any means, a comprehensive treatise. The arrangement of the work is excellent; the chapter on the Soil is especially to be commended, as is also that on Hygiene of School.

The chapter on Antiseptics, Disinfectants and Deodorants, is good, except in one particular ; it gives no method of liberating chlorine gas for purposes of disinfection after the presence of infectious disease. This is, in our opinion, the only true disinfectant gas for such purposes.

On the whole, the book is to be commended as a text-book, but needs considerable elaboration on the part of the instructor.

DeL. R.

